# The protein tyrosine kinase SYK regulates the alternative p38 activation in liver during acute liver inflammation

**DOI:** 10.1038/s41598-019-54335-3

**Published:** 2019-11-28

**Authors:** Bo-Ram Bang, Kyung Ho Han, Goo-Young Seo, Michael Croft, Young Jun Kang

**Affiliations:** 1 Department of Immunology and Microbial Science, The Scripps Research Institute, La Jolla, CA 92037 USA; 2 Department of Chemistry, The Scripps Research Institute, La Jolla, CA 92037 USA; 3 Department of Medicine, Division of Gastrointestinal and Liver Diseases, Keck School of Medicine, University of Southern California, Los Angeles, CA 90033 USA; 4 Division of Developmental Immunology, La Jolla Institute for Immunology, La Jolla, CA 92037 USA; 5 Division of Immune Regulation, La Jolla Institute for Immunology, La Jolla, CA 92037 USA; 6 Molecular Medicine Research Institute, Sunnyvale, CA 94085 USA

**Keywords:** Kinases, Stress signalling

## Abstract

Two distinct p38 signaling pathways, classical and alternative, have been identified to regulate inflammatory responses in host defense and disease development. The role of alternative p38 activation in liver inflammation is elusive, while classical p38 signaling in hepatocytes plays a role in regulating the induction of cell death in autoimmune-mediated acute liver injury. In this study, we found that a mutation of alternative p38 in mice augmented the severity of acute liver inflammation. Moreover, TNF-induced hepatocyte death was augmented by a mutation of alternative p38, suggesting that alternative p38 signaling in hepatocytes contributed more significantly to the pathology of acute liver injury. Furthermore, SYK-Vav-1 signaling regulates alternative p38 activation and the downregulation of cell death in hepatocytes. Therefore, it is suggested that alternative p38 signaling in the liver plays a critical role in the induction and subsequent pathological changes of acute liver injury. Collectively, our results imply that p38 signaling in hepatocytes plays a crucial role to prevent excessive liver injury by regulating the induction of cell death and inflammation.

## Introduction

Mitogen-activated kinase (MAPK) signaling plays an essential role in inflammation and host defense in immunity by regulating the production of pro-inflammatory cytokines and chemokines^1–5^. p38, a member of the MAPK family, plays a key role for the induction of pro-inflammatory cytokines, chemokines and several other inflammatory molecules in host defense and inflammatory disease development^4,6–9^. Therefore, it has been postulated that treatment with p38 inhibitor compounds would be beneficial for inflammatory disease treatment, since p38 inhibitors blocked the expression of inflammatory cytokines and ameliorated the development of inflammatory diseases in animal studies^10–12^. However, the *in vivo* use of such inhibitors caused severe liver cytotoxicity. Although administration of a p38 inhibitor reduced significantly the levels of inflammatory biomarkers in clinical studies, they also showed severe hepatotoxicity, including increased aminotransferase levels in sera^10^. Despite the liver toxicity issue, further development of strategies that target p38 activation is anticipated due to the enzyme’s potential as an anti-inflammatory target. Therefore, a better understanding of p38 signaling and its role in inflammation and cell death may provide new avenues for the development of clinically acceptable therapeutic strategies.

In the p38 signaling pathway, stimuli such as ligand-receptor interaction or environmental stresses induce the activation of several mitogen-activated protein kinase kinase kinases (MKKKs) that further phosphorylate and activate the dual-specificity kinases mitogen-activated protein kinase kinase 3 (MKK3) and MKK6. The Tyr180 and Thr182 residues in p38 are phosphorylated by MKK3/6^5^. Unlike this classical (or canonical) pathway, alternative p38 signaling is independent of MKK3/6 activity. TCR-triggered Zap70 phosphorylates p38 on Tyr323, which in turn autophosphorylates Thr180, but not Tyr182^13,14^, implying different substrate specificity. Studies using knock-in mice with a Tyr-to-Phe substitution at amino acid residue 323 of p38αβ (p38αβ^Y323F^, p38YF) demonstrated that T cells from mutant mice had defects in TCR-mediated proliferation and Th1/Th17 skewing and exhibited delayed onset and reduced severity of autoimmune diseases. TCR-mediated activation of CD4^+^ tumor-infiltrating lymphocytes also results in alternative p38 activation and production of pro-tumorigenic factors^13,15–17^.

Our previous study demonstrated that the activation of classical p38 signaling regulates acute liver inflammation in a tissue-specific manner, and ablation of p38 in the liver results in liver toxicity^9^, suggesting that liver toxicity by p38 inhibitors may be a result of the inhibition of protective activity of p38 in liver. However, it has not been elucidated how alternative p38 activation regulates the induction of inflammation and cell death in liver.

Here, we demonstrate that alternative p38 activation regulates the induction of liver cell death by a unique mechanism during liver injury. We found that mice carrying a mutation in alternative p38 signaling (p38YF) showed increased inflammation in a model of Concanavalin A (Con A)-induced acute liver injury. Our results indicate that mutation of alternative p38 signaling in liver is associated with the induction of cell death, suggesting a protective role of the alternative p38 signaling pathway. Alternative p38 activation, however, regulates the induction of inflammatory responses in T and NKT cells in such a way that a p38YF mutation results in reduced liver inflammation. Therefore, we hypothesized that alternative p38 signaling regulates the induction of cell death in hepatocytes via a distinct mechanism from the classical p38 activation. Mutation in the alternative p38 signaling in the liver induced apoptosis and necrotic cell death directly in hepatocytes, implying a distinct function of this pathway in liver pathology. TNF-α-induced activation of alternative p38 is dependent on the SYK-Vav-1 signaling pathway in hepatocytes, leading to the regulation of Gadd45α (the growth, arrest and DNA damage-inducible 45α protein) expression that is crucial in survival and in induction of cell death. Our observations support the notion that alternative p38 signaling in liver plays a critical protective role in the induction and pathological changes of acute liver injury via a previously unidentified distinct mechanism in hepatocytes. Our study will facilitate a better understanding of the novel mechanism of p38 activation in hepatocytes and provide clues to avoiding the severe liver toxicity caused by p38 inhibitors as well as establish an improved strategy for the treatment of diseases characterized by inflammatory processes.

## Results

### Mutation in alternative p38 signaling augments Con A-induced liver injury

We showed previously that the classical p38 signaling pathway regulates inflammatory responses either positively or negatively depending on the cell or tissue types^9^. Pathological changes associated with liver injury were distinctly different between T cell- and liver-specific classical p38-deficient mice. Liver injury was significantly reduced by deletion of p38 in T cells, while ablation of liver p38 aggravated Con A-induced liver injury. Classical p38 signaling in hepatocytes, however, did not contribute to the induction of cell death directly *in vitro*.

**Figure 1:**
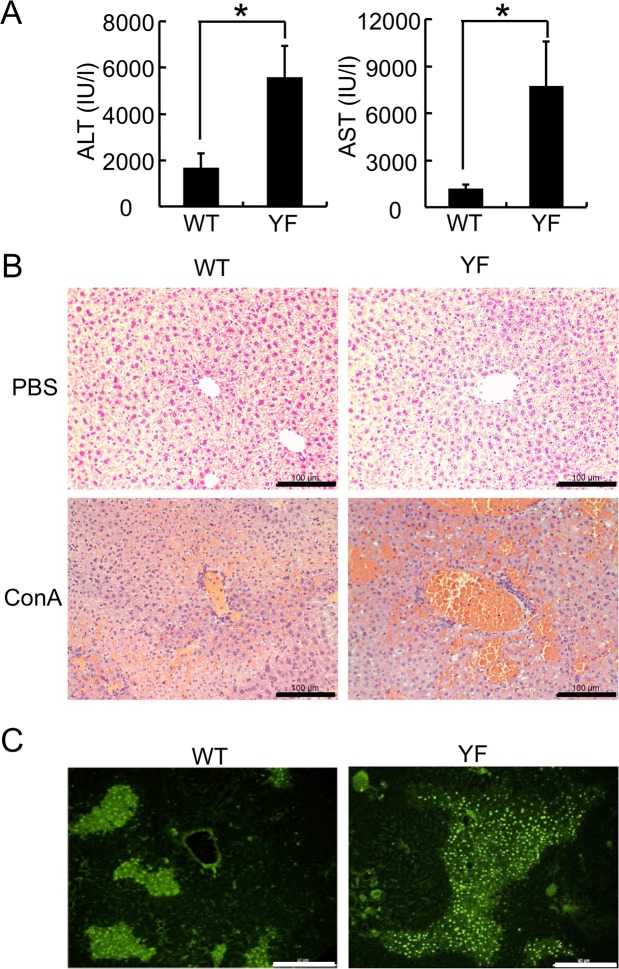
Con A-induced liver damage is augmented in p38YF mice. (**A**) WT or p38YF mice were injected with Con A intravenously. After 8 h, serum samples were collected, and ALT and AST levels were measured (n = 6). (**B**) H & E staining of liver specimens from PBS- or Con A-injected mice. Scale bars = 100 μm. (**C**) TUNEL staining of liver tissues from Con A-injected WT or p38YF mice. Scale bars = 40 μm. Data represent means ± s.d.; *p < 0.001. Results shown are representative of 2–3 independent experiments.

To investigate the role of the alternative p38 signaling pathway in liver inflammation, we used a Con A–induced acute liver injury model that is mediated largely by T and NKT cells^18,19^. WT and p38YF mice were injected with Con A intravenously, and serum aminotransferase levels were measured. Alanine aminotransferase (ALT) and aspartate aminotransferase (AST) levels in p38YF mice were increased significantly compared with WT mice (Fig. 1A). Additionally, liver pathology characterized by infiltration of inflammatory cells, liver hemorrhage, and cell death was more severe in p38YF mice than WT mice (Fig. 1B). TUNEL assay indicated that apoptosis was significantly induced in the liver tissue of p38YF mice (Fig. 1C). Collectively, these results suggest that the alternative p38 signaling pathway plays a protective role in Con A-induced acute liver injury.

### Alternative p38 signaling promotes the expression of inflammatory cytokines in T and NKT cells

**Figure 2:**
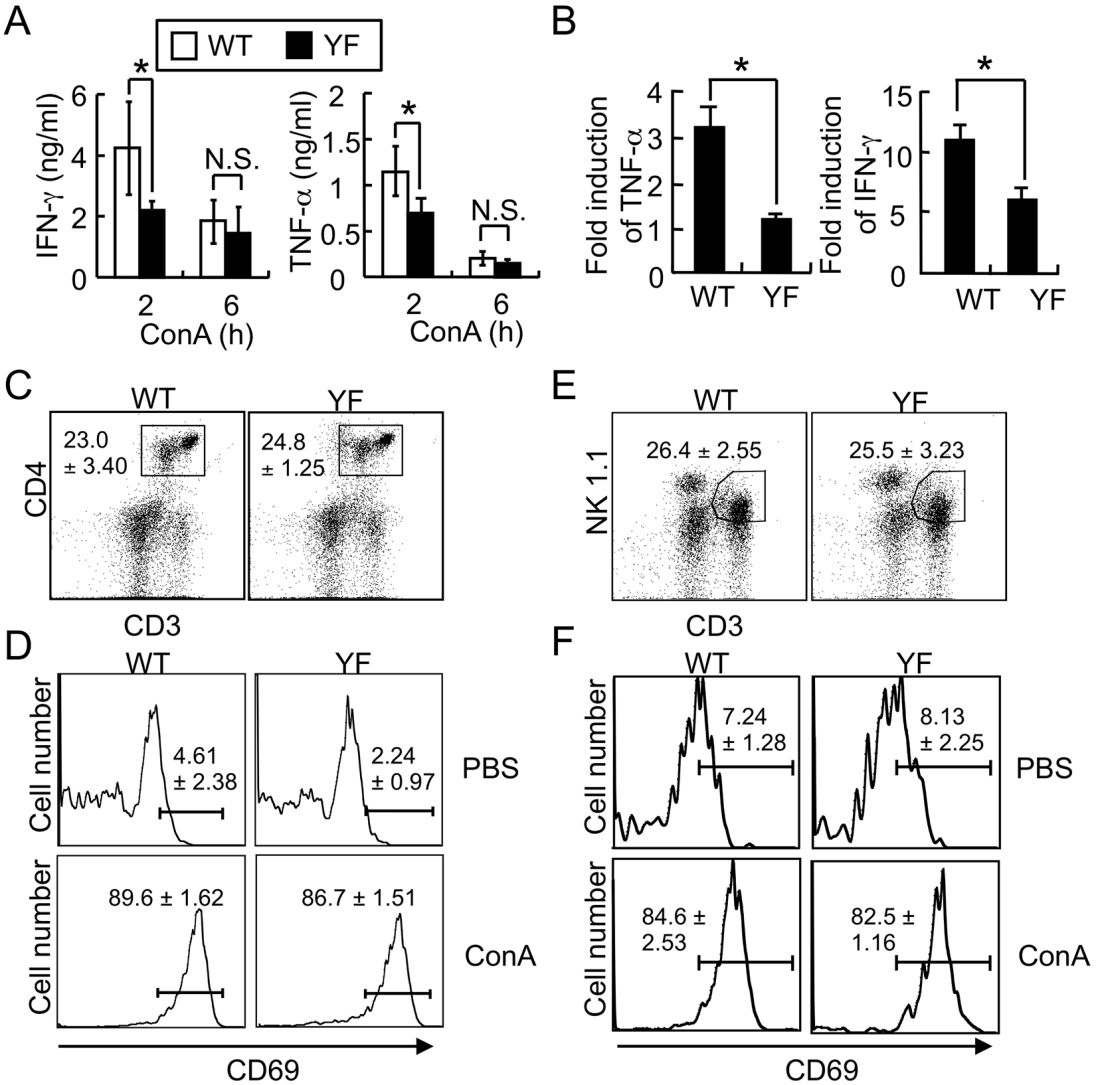
Production of inflammatory cytokines is reduced by mutation of the alternative p38 signaling *in vivo*. (**A**,**B**) WT or p38YF mice received Con A intravenously. (**A**) Serum IFN-γ and TNF-α levels were determined by ELISA or (**B**) expression of cytokines in liver leukocytes was determined by qPCR analysis after 2 or 6 h of Con A administration (n = 6). Fold induction was calculated by comparing to the liver tissues of PBS-injected mice. (**C**–**F**) Flow cytometry analysis of T and NKT cells in liver. Liver leukocytes from WT or p38YF mice (n = 4) were co-stained with anti-mouse CD3, CD4, NK1.1, and CD69 antibodies at 4 h after Con A administration. The percentages of T cells (CD3^+^ CD4^+^) and NKT cells (CD3^+^NK1.1^+^) in the total population of liver leukocytes (C & E), and the percentages of CD69-positive cells from CD3^+^ CD4^+^ or CD3^+^NK1.1^+^ population (**D**,**F**) are shown. Data shown are means ± s.d.; *p < 0.05 and **p < 0.01. N.S., not significant. Results shown are representative of 2–3 independent experiments.

Because T and NKT cells play an important role in Con A–induced liver damage^20–22^, we further tested whether alternative p38 signaling negatively regulates the production of inflammatory cytokines in T and NKT cells. We found that TNF-α and IFN-γ levels in sera and expression of cytokines in liver leukocytes were significantly reduced in p38YF mice compared with WT mice after Con A administration (Fig. 2A,B), indicating the regulatory role of alternative p38 for the expression of inflammatory cytokines by T and NKT cells in acute liver injury. Next, we examined whether there were distinct differences in immune cell composition and activation marker expression in livers between WT and p38YF mice. The number of CD3^+^CD4^+^ T cells and CD3^+^NKT1.1^+^ NKT cells, and the expression of the activation marker CD69 were comparable in hepatic leukocytes from Con A-administered WT and p38YF mice^23–26^ (Fig. 2C–F). Collectively, alternative p38 activation regulates the expression of inflammatory cytokines in Con A-induced acute liver injury.

We further examined the expression of inflammatory cytokines in liver T cells and NKT cells *in vitro*. The production and expression of IFN-γ and TNF-α were significantly reduced in p38YF T cells (stimulated with anti-CD3/CD28 Abs, Fig. S1A,B) and p38YF NKT cells (stimulated with NKT cell-specific ligand α-galactosylceramide (α-GalCer), Fig. S1C,D) when compared with WT cells. These results suggest that alternative p38 activation in T and NKT cells positively regulates the expression of inflammatory cytokines.

### Alternative p38 signaling in liver plays a protective role in Con A-induced liver injury

**Figure 3:**
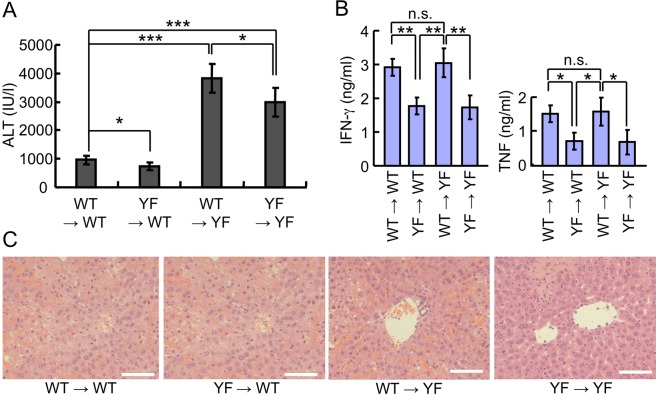
Alternative p38 activation in liver regulates the induction of acute liver injury. BM chimeric mice were generated as the following donor → recipient combinations: WT → WT, WT → p38YF, p38YF → WT, and p38YF → p38YF. After 6 weeks, mice were injected with Con A intravenously (n = 4). (**A**) Serum ALT levels were measured after 12 h, and (**B**) IFN-γ and TNF-α levels in blood samples were analyzed after 2 h. (**C**) H & E staining of liver tissues. Scale bars = 100 μm. Data represent means ± s.d., *p < 0.05; **p < 0.01; ***p < 0.005, and n.s., not significant.

Although production of inflammatory cytokines by T and NKT cells was significantly reduced by the mutation of alternative p38, the severity of Con A-induced liver injury was greater in p38YF mice. Thus we hypothesized that the alternative p38 pathway in hepatocytes plays a more significant role in Con A-induced liver injury. Since tissue-specific p38YF mice are not available, we generated bone marrow (BM) chimeric mice of the following donor → recipient combinations to test our hypothesis: WT → WT, WT → p38YF, p38YF → WT, and p38YF → p38YF. We found that the serum ALT level was reduced in p38YF → WT mice compared with WT → WT, while ALT levels were higher in WT → p38YF and p38YF → p38YF mice compared with WT → WT (Fig. 3A). The ALT level in WT → p38YF was higher than in p38YF → p38YF mice. Additionally, serum levels of inflammatory cytokines in Con A-injected mice were reduced in p38YF → WT or p38YF → p38YF mice compared with WT → WT or WT → p38YF mice (Fig. 3B). Production of cytokines was comparable between WT → WT and WT → p38YF, or p38YF → WT and p38YF → p38YF mice, supporting the role of the alternative p38 pathway in the activation of T and NKT cells. The severity of liver inflammation upon histopathological examination showed a similar trend to that of ALT levels; WT → p38YF > p38YF → p38YF > WT → WT > p38YF → WT (Fig. 3C). This result indicated that p38YF hepatocytes are more sensitive to the effects of stimuli such as inflammatory cytokines than WT cells, although the levels of inflammatory cytokines are lower in p38YF mice, leading to the increased induction of cell death.

### Alternative p38 activation regulates TNF-α-induced cell death in hepatocytes

**Figure 4:**
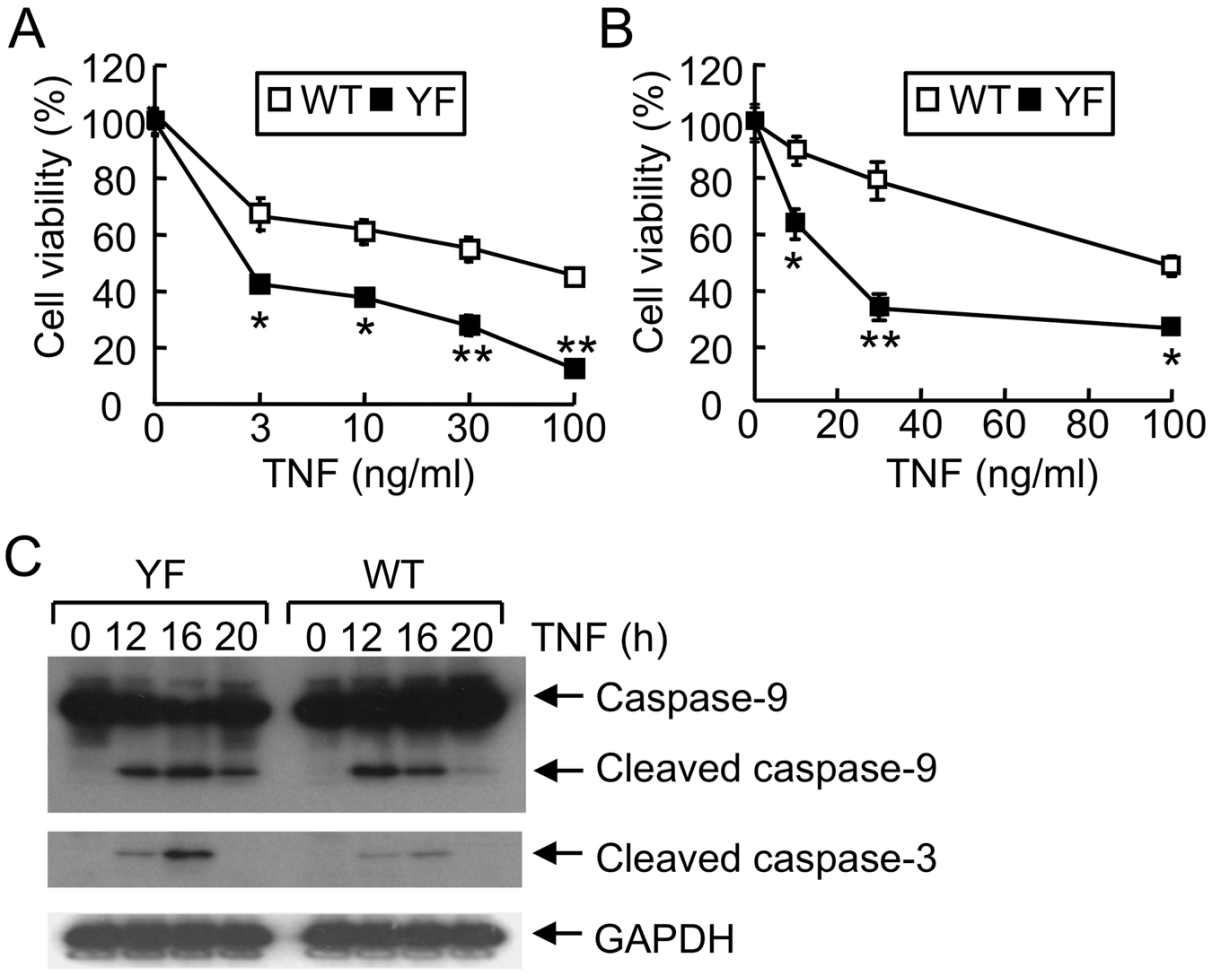
Alternative p38 signaling protects hepatocytes from TNF-α-induced cell death. (**A**,**B**) Hepatocytes from WT or p38YF mice (**A**), or WT or p38YF Hepa 1–6 cells (**B**) were incubated with actinomycin D (ActD) and TNF-α. Cell viability was assessed after 24 h by XTT assay (n = 6). (**C**) WT or p38YF Hepa 1–6 cells expressing wildtype or mutant p38 were treated with TNF-α (30 ng/ml). Cell lysates were prepared and the activation of caspases was analyzed by immunoblotting using anti-cleaved caspase-3 or caspase-9 antibodies. GAPDH was visualized as an internal control. Data represent means ± s.d.; *p < 0.05; **p < 0.01; and n.s., not significant. Results shown are the representative of 2–3 independent experiments. Full-length blot images are shown in the Supplementary Information (Fig. S5).

Based upon our observation that apoptosis was increased in liver of p38YF mice (Fig. 1C), we examined whether alternative p38 signaling in hepatocytes contributed to the regulation of cell death. TNF-α plays a significant role in acute liver damage^19,27^, thus the effect of the p38YF mutation of p38 signaling on TNF-α-induced death of hepatocytes was examined. Primary hepatocytes from WT or p38YF mice, or Hepa 1–6 hepatocyte cell lines that constitutively express WT p38 or the p38YF mutant, were treated with TNF-α. We found that induction of cell death was significantly increased in p38YF hepatocytes compared with WT cells (Fig. 4A,B), a finding which has not been reported previously. Unlike hepatocytes where classical p38 signaling is comparable between WT and classical p38-deficient hepatocytes^9^, TNF-α-induced cleavage of caspases was enhanced in p38YF cells (Fig. 4C), indicating that alternative p38 regulates the induction of apoptosis. Mutation in the alternative p38 signaling pathway in hepatocytes induced apoptosis and necrotic cell death more significantly than WT p38 activation after TNF-α treatment, implying a distinct function of this pathway in liver pathology (Fig. S2).

### Expression of Gadd45α is negatively regulated by alternative p38 in hepatocytes

**Figure 5:**
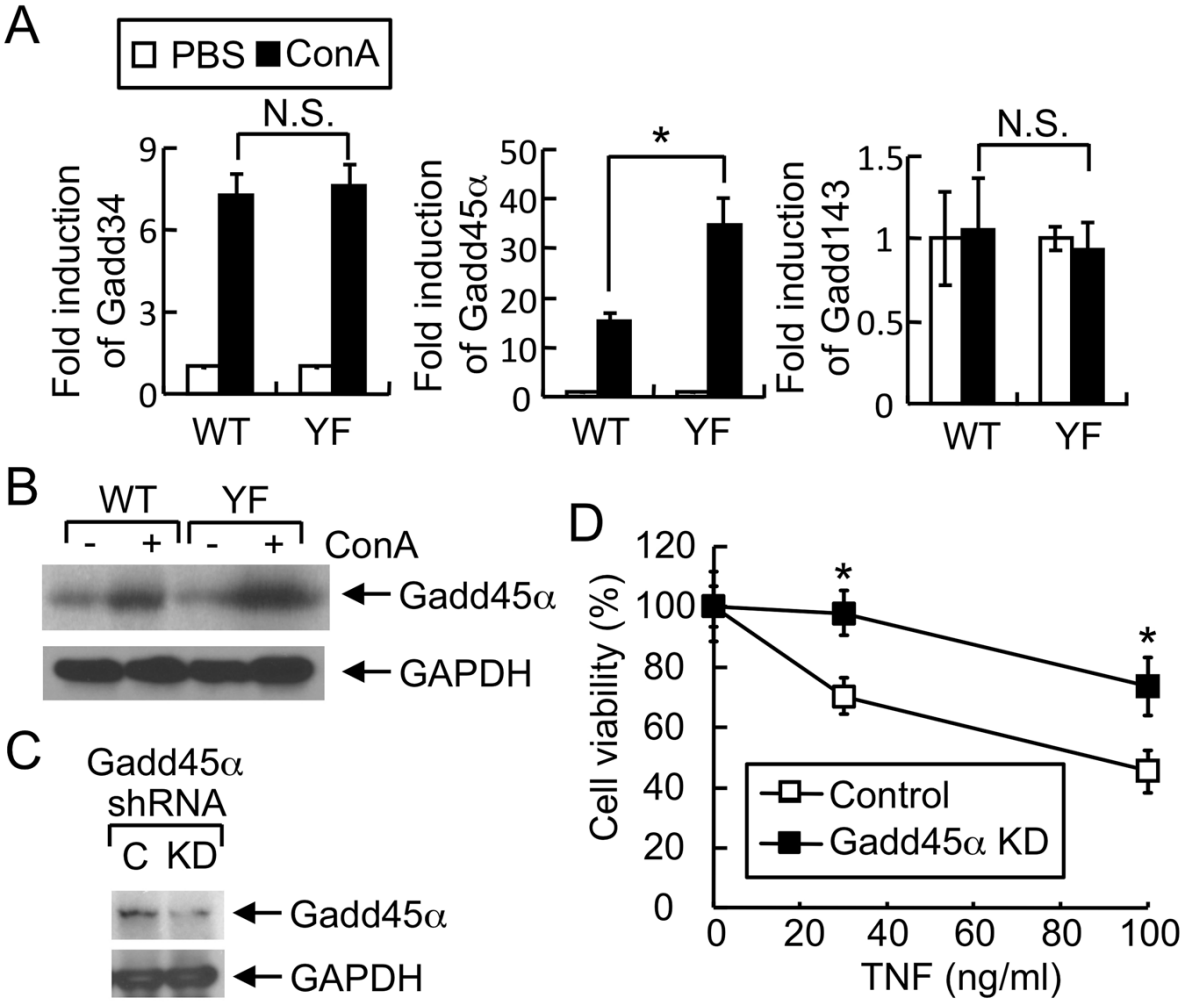
Alternative p38 activation regulates the induction of Gadd45α for the survival of hepatocytes in acute liver injury. (**A**) Expression of Gadd family members in liver tissues was determined by qPCR analysis after 6 h of intravenous Con A injection (n = 4). Fold induction was calculated by comparing with the liver tissues of PBS-injected mice. (**B**) Induction of Gadd45α in liver of Con A-injected mice. WT or p38YF mice were administered Con A or not, and liver lysates were prepared after 4 h. Expression of Gadd45α was analyzed by immunoblotting using anti-Gadd45α antibody. GAPDH levels were examined as an internal loading control. (**C**,**D**) Gadd45α contributes to TNF-α-induced death of hepatocytes. Control (**C**) or Gadd45α KD Hepa 1–6 cells were generated by infecting lentiviruses encoding control or Gadd45α shRNA. Knockdown of Gadd45α was confirmed by immunoblotting (**C**), and cells treated with ActD and TNF-α (n = 6). Cell viability was measured by XTT assay after 24 h. *p < 0.05. Results shown are the representative of 2–4 independent experiments. Full-length blot images are shown in the Supplementary Information (Fig. S6).

Next, we examined the expression of Gadd (the growth, arrest and DNA damage-inducible protein) members such as Gadd34, Gadd45α, and Gadd143 that play a role in apoptosis^28,29^, in the liver tissues of Con A-injected mice. Expression of Gadd45α was significantly increased in the liver tissue of Con A-injected p38YF mice (Fig. 5A,B), suggesting that alternative p38 activation regulates the expression of Gadd45α in liver. Expression of Gadd143 was not induced by Con A, while Con A-induced Gadd34 expression was comparable between livers of WT and p38YF mice (Fig. 5A). We next generated Gadd45α KD Hepa 1–6 cells to determine the involvement of Gadd45α in TNF-α-induced cell death. We found that TNF-α-induced cell death was significantly lower in Gadd45α KD hepatocytes compared with control cells (Fig. 5C,D). Collectively, our results suggest that alternative p38 activation in hepatocytes regulates the expression of Gadd45α and thereby the survival of hepatocytes in liver injury.

### Activation of alternative p38 is independent of MKK-mediated MAPK activation in hepatocytes

We next addressed the mechanism by which alternative p38 activation influences TNFR signaling in hepatocytes. Degradation of IκB-α and phosphorylation of MAPKs such as p38 (Thr180/Tyr182), ERK, and JNK were comparable between TNF-α-treated WT and p38YF hepatocytes, whereas phosphorylation of alternative p38 (Tyr323) was only detected in WT cells (Fig. S3). Activation of classical p38 and JNK can be regulated directly by the upstream mitogen-activated kinase kinases such as MKK3/6 and MKK4, respectively. We found that phosphorylation of MKK3/6 and MKK4 was not affected by mutation of the alternative p38. The expression of TAB1 protein was not affected by alternative p38 signaling in hepatocytes. Collectively, our results suggest that alternative p38 activation regulates the induction of cell death in liver independently of the classical MKK-mediated MAPK pathway.

### SYK-mediated signaling regulates the activation of alternative p38 in hepatocytes

**Figure 6:**
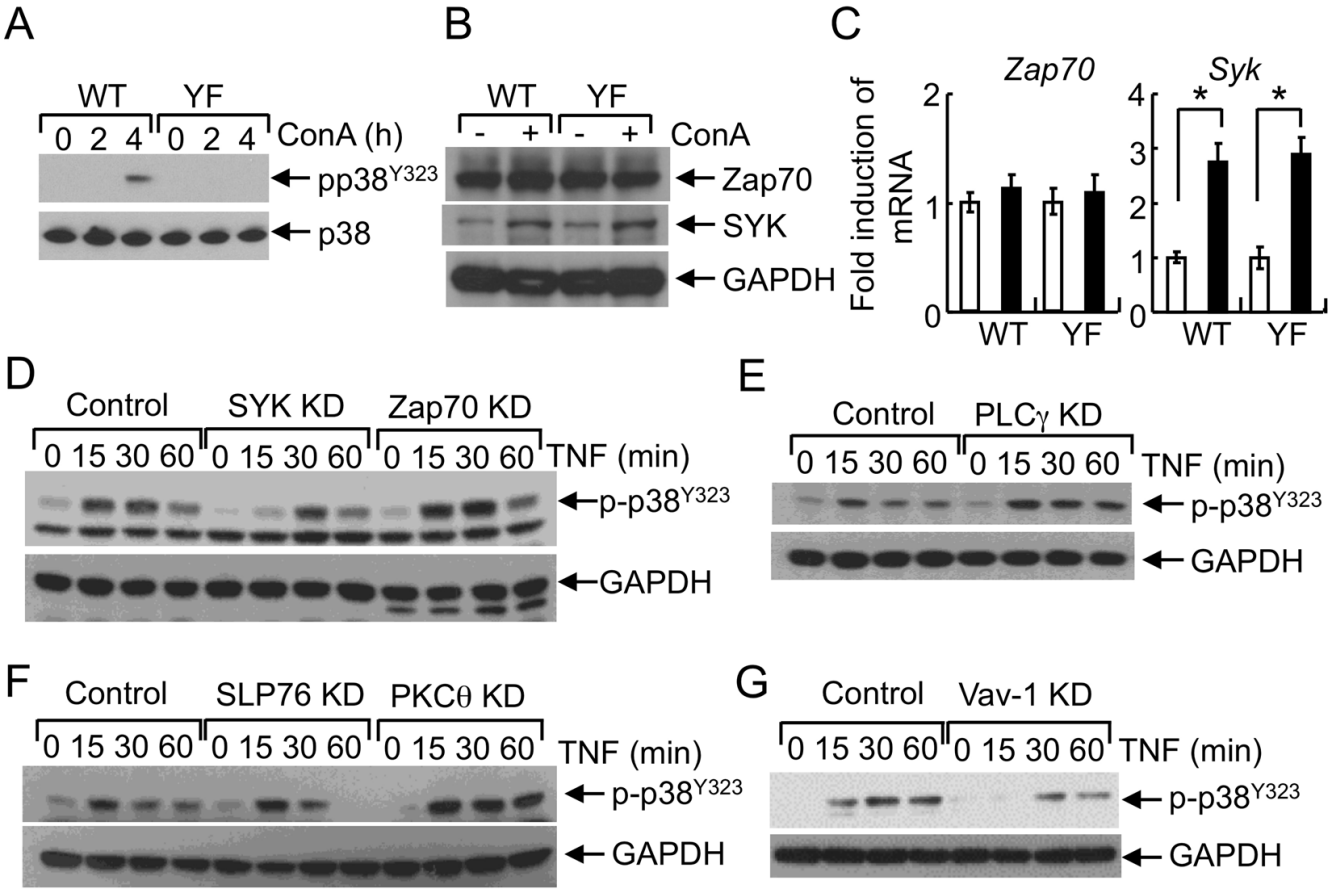
Involvement of signaling components in alternative p38 activation in hepatocytes. (**A**–**C**) Phosphorylation of alternative p38 and expression of SYK in liver. WT or p38YF mice were injected with Con A intravenously, and liver tissues were obtained after 2 or 4 h (**A**) or 4 h (**B**,**C**) to prepare cell lysates and RNA. (**A**,**B**) phosphorylation of alternative p38 and protein levels of Zap70 and SYK were analyzed by immunoblotting using anti-pp38^Y323^, anti-Zap70 or SYK antibodies, respectively. (**C**) Fold induction of Zap70 and SYK mRNA was determined by qPCR analysis (n = 4) Fold induction was calculated by comparing with the liver tissues of PBS-injected mice. (**D**–**G**) Cells were treated with TNF-α and cell lysates were prepared at indicated times. Phosphorylation of p38^Y323^ was detected by immunoblotting. Unphosphorylated p38 and GAPDH levels were determined as a loading control. *p < 0.01. Results shown are the representative of 2–3 independent experiments. Full-length blot images are shown in the Supplementary Information (Figs. S7 and 8).

We further examined the mechanism of alternative p38 activation in hepatocytes. A previous study demonstrates that Zap70, a tyrosine kinase, regulates the activation of alternative p38 signaling in T cells^13^. Thus, we examined the expression of protein tyrosine kinases that may regulate the activation of alternative p38 signaling in hepatocytes. Phosphorylation of alternative p38 (p38^Y323^) was detected in the liver tissues of Con A-administered WT mice, not in those of YF mice (Fig. 6A). Expression of Zap70 was not induced in the liver tissues of Con A-injected WT and p38YF mice. However, another tyrosine kinase, spleen tyrosine kinase (SYK) was increased by Con A administration (Fig. 6B,C). SYK is expressed in hepatocytes^30^ and plays a crucial role in acute liver injury, steatosis and inflammation^31,32^. Additionally, activation of SYK by TNF-α induces MAPK activation and apoptosis^33^. Therefore, we examined whether SYK regulates the activation of alternative p38 signaling in hepatocytes. To this end, we generated control, Zap70- or SYK KD hepatocytes by infecting Hepa 1–6 cells with lentiviruses encoding control, Zap70, or SYK shRNAs (Fig. S4). We found that TNF-α-induced phosphorylation of p38 (Tyr323) was comparable between control and Zap70 KD cells, whereas knockdown of SYK reduced p38 (Tyr323) phosphorylation (Fig. 6C), indicating that SYK is an upstream component of the alternative p38 signaling pathway. Of the several intermediate molecules implicated in relaying SYK-mediated downstream signaling, Vav-1, phospholipase Cγ (PLCγ), the SH2 domain-containing leukocyte protein 76 (SLP76) can associate directly with SYK^34^, which further regulates protein kinase Cθ (PKCθ) that can then activate p38. We determined the involvement of signaling intermediates by generating gene KD hepatocytes by infecting Hepa 1-6 cells with lentiviruses encoding shRNAs^35^ (Fig. S5). We found that Vav-1, but not PLCγ, SLP76, and PKCθ, influences TNF-induced phosphorylation of alternative p38 in liver (Fig. 6D–F), suggesting that SYK → Vav−1 signaling regulates the alternative p38 activation in hepatocytes.

### Inhibition of SYK-mediated signaling induces cell death in hepatocytes

**Figure 7:**
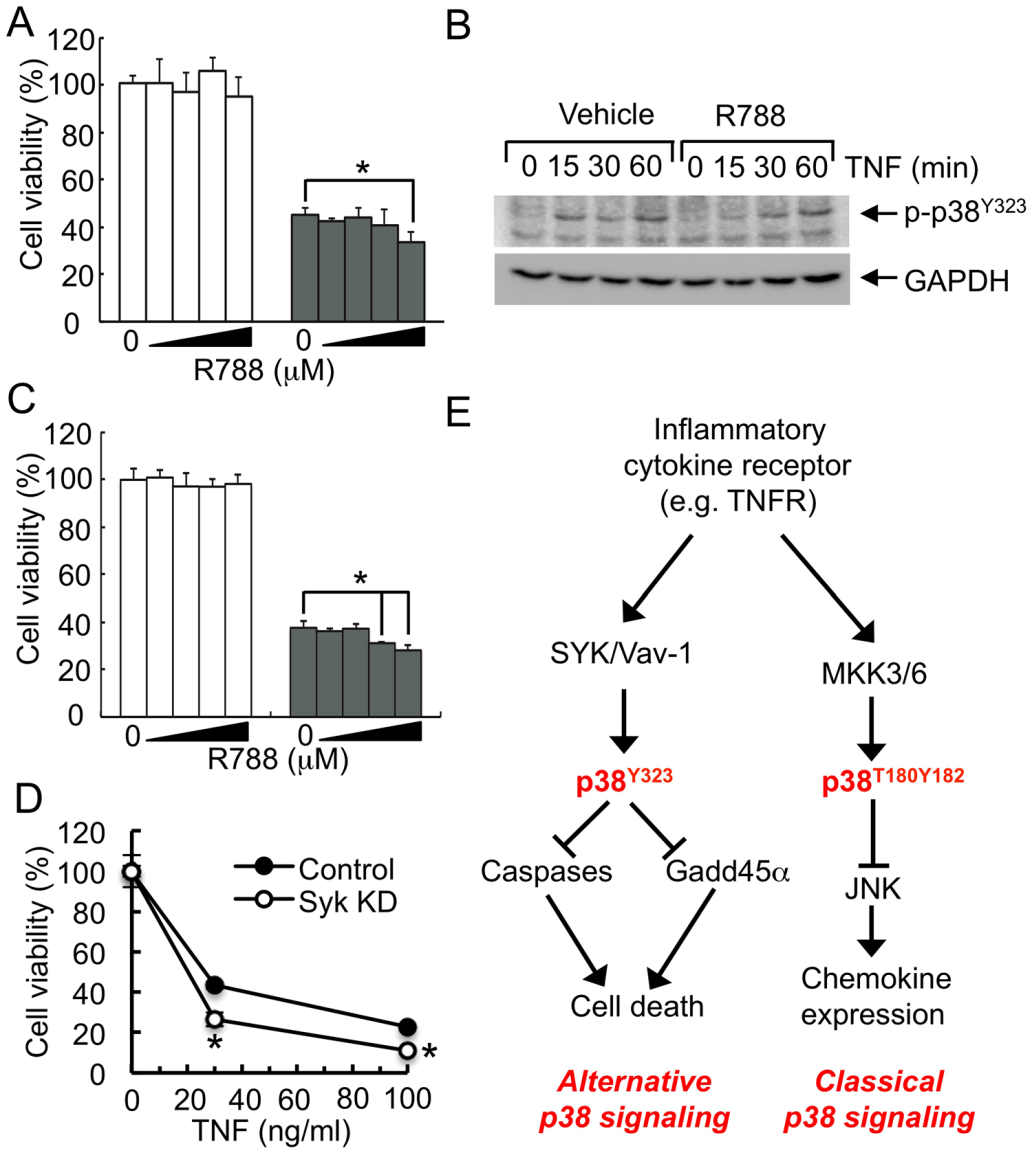
Inhibition of SYK by R788 induces cell death in hepatocytes. (**A**,**B**) Hepa 1–6 cells were cells treated with ActD and R788 (0, 1‚ 3, 10, or 30 μM) for 1 hour, and treated with medium (open barss) or mouse TNF-α (closed bars) (n = 6). (**A**) Cell viability was measured by XTT assay after 24 h and (**B**) cell lysates were prepared at indicated times to analyze the phosphorylation of p38 (Tyr323) by immunoblotting using anti-phospho-p38 (Tyr323) antibody. GAPDH levels were examined as an internal loading control. (**C**) Viability of R788-treated HepG2 cells was measured as described in (**A**) except cells were treated with medium (open bars) or human TNF-α (closed bars). (**D**) Control or SYK KD Hepa 1–6 cells were treated with ActD and TNF-α (n = 6). Cell viability was measured by XTT assay after 24 h. *p < 0.05. Results shown are the representative of 2–4 independent experiments. (**E**) Distinct p38 signaling pathways protect liver from excessive inflammation and cell death in acute liver inflammation. In alternative p38 activation pathway, TNFR-induced activation of SYK → Vav−1 signaling regulates the activity of alternative p38 to inhibit the caspase- or Gadd45α-mediated induction of cell death. In contrast, MKK-dependent activation of classical p38 negatively regulates the activation of JNK to suppress the recruitment of inflammatory immune cells in liver. Full-length blot images are shown in the Supplementary Information (Fig. S9).

A highly specific SYK inhibitor, R788 or fostamatinib, has been shown to inhibit the development of experimental arthritis^36,37^, and to be effective in the treatment of patients with rheumatoid arthritis (RA)^38,39^. However, side effects included diarrhea, neutropenia, increased blood pressure, and ALT elevation, which is associated with the higher doses of R788. Thus, we further tested whether inhibition of SYK affects the activation of alternative p38 and subsequent induction of death in hepatocytes, which may be responsible for the elevation of ALT in patients. In Hepa 1–6 cells, R788 potentiated TNF-α-induced cell death at high concentrations, and reduced or delayed the activation of alternative p38, indicating that inhibition of SYK by R788 increases the induction of cell death by inhibiting the activation of alternative p38 in hepatocytes (Fig. 7A,B). Additionally, high doses of R788 also induced cell death of TNF-α-treated human hepatocyte HepG2 cells (Fig. 7C). Knocking-down of SYK reduced the phosphorylation of p38 (Tyr323) and increased cell death in TNF-α-treated hepatocytes (Figs. 6C and 7D). Therefore, our data suggest that SYK regulates the activation of the alternative p38 pathway and high concentration of SYK can potentiate hepatocyte death that may be responsible for the elevated liver enzyme levels in patients treated with R788.

In conclusion, our results suggest a protective role of alternative p38 signaling in liver in the induction and subsequent pathological changes of acute liver injury. The SYK → Vav-1 signaling regulates the activation of alternative p38 for the regulation of cell death induction via Gadd45α expression in hepatocytes. Our study will provide clues to avoiding the severe liver toxicity caused by p38 inhibitors and help establishing an improved strategy for the treatment of inflammatory diseases.

## Discussion

Two distinct p38 signaling pathways, classical and alternative, have been identified as regulating inflammatory responses. The classical p38 signaling pathway promotes pro-inflammatory responses in immune cells associated with inflammation and disease development. In addition to its role for the development of inflammatory diseases, activation of classical p38 signaling enhances host defense to microbial infection such as *C. rodentium* by limiting the bacterial burden and regulating the responses of T cells and intestinal epithelial cells^8,9^. In contrast, alternative p38 activation regulates T cell activation independently of upstream MKK3/6 activation and plays a role in limiting microbial infection and disease development^13,15,16^. We showed previously that autoimmune-mediated acute liver injury was alleviated by T cell-specific ablation of classical p38 in mice, indicating the pro-inflammatory role of p38 in T cells, while liver-specific ablation of classical p38 resulted in augmented liver damage, suggesting a protective role of liver p38 activation in liver injury. The mechanism by which alternative p38 signaling plays a role in liver inflammation has been unclear. In this study, we elucidate the role of alternative p38 signaling in acute liver injury and further identify the mechanism that regulates this pathway in the induction of cell death in liver.

In immune-mediated liver injury such as Con A-induced hepatitis, pro-inflammatory cytokines such as IFN-γ and TNF-α produced by T and NKT cells play a crucial role in the development of the pathological changes observed in liver^40,41^. We observed that a mutation of alternative p38 augmented the acute liver injury compared with WT mice, suggesting a protective role for alternative p38. However, we noted that inflammatory cytokine levels in serum and the expression of cytokines by liver leukocytes were reduced in p38YF mice, and that the mutation of alternative p38 signaling caused a reduction of TNF-α and IFN-γ production by T and NKT cells *in vitro*. In line with this, previous studies suggested that alternative p38 activation regulates T cell signaling in host defense and autoimmune disease development^13,15–17^. However, our results suggest the cell-specific role of alternative p38 signaling in acute liver inflammation as BM transplantation experiment indicates that p38YF hepatocytes are more sensitive to the effects of stimuli such as inflammatory cytokines than WT cells.

Alternative p38 signaling regulates the induction of cell death in hepatocytes as p38YF mutation increased apoptosis by enhancing caspase activities and Gadd45α expression, which is not observed in classical p38-deficient hepatocytes^9^. Gadd45α can regulate cell cycle arrest, DNA repair, and cell survival, and the alternative p38 activation pathway in T cells^13,42–45^. Despite some controversies, Gadd45α is known to play a role in regulating the induction of apoptosis^46,47^. KD of Gadd45α improved the survival of hepatocytes treated with TNF-α. Therefore, unlike signaling in T cells and dendritic cells in which Gadd45α regulates the activation of alternative p38^13,45^, alternative p38 activation regulates the expression of Gadd45α in hepatocytes in liver inflammation.

Previous studies showed that TCR-mediated activation of alternative p38 signaling is mediated by the TCR-proximal tyrosine kinase Zap70, but not the adaptor protein LAT^13^. TNF-α-dependent activation of alternative p38 signaling, however, requires SYK that is a receptor-proximal tyrosine kinase but not Zap70 in hepatocytes. SYK displays diverse biological functions including immune recognition, vascular development and platelet activation^34^. Previous studies have identified a pathogenic role of SYK for the development of renal interstitial fibrosis, RA, leukemia, and liver fibrosis^48–51^, suggesting a potential therapeutic intervention point for the treatment of such diseases. Our result indicates that SYK-Vav1 regulates the activation of alternative p38 signaling in liver.

Despite our finding that alternative p38 activation is dependent on a SYK-Vav-1 cascade for the negative regulation of caspase- or Gadd45α-mediated cell death induction in hepatocytes, we cannot rule out the involvement of other signaling molecules such as kinases or phosphatases for the cross-talk with alternative p38 signaling in hepatocytes in liver disease. Therefore, further studies are needed to better understand the role of alternative p38 signaling, which may provide an option for the development of therapeutic strategies for liver disease treatment.

In addition to its role as a mediator of immunoreceptor signaling in macrophages, neutrophils, mast cells, and B cells, activation of SYK is important for TNF-α-induced cytokine inflammatory production in synoviocytes from RA patients^49^. R788, a specific SYK inhibitor, exhibits potent anti-inflammatory activity, suggesting a role for SYK inhibition in the treatment of RA^36–39^. However, an elevation of liver enzyme level is also observed in patients with high doses of R788 treatment. In this study, we found that SYK-dependent signaling regulates alternative p38 activation that protects the induction of cell death in liver, suggesting that a side effect of ALT elevation may be a result of reduced alternative p38 activity by SYK inhibition.

In this report, we suggest the distinctive role of p38 in liver pathology. However, it is not clear how alternative p38 regulates the downstream signaling independently of the classical p38 pathway. Here, we suggest that SYK-dependent pathway selectively regulates the activation of alternative p38 pathway in liver. In liver diseases, SYK contributes to the development of alcoholic liver disease and liver fibrosis^31,51^, suggesting that SYK can be a potential therapeutic target for liver diseases. Therefore, further studies are needed to better understand the detailed mechanism of SYK in acute liver inflammation mediated by immune cell activation.

Taken together with our previous work concerning the role of classical p38 signaling in acute liver injury, our findings suggest a tissue-specific function of alternative p38 in inflammation. Inhibition or ablation of classical p38 results in JNK activation, induction of chemokine expression, and immune cell recruitment, leading to increased cell death in liver^9^ (Fig. 7E), suggesting the protective role of classical p38 in acute liver inflammation. In contrast, SYK-Vav-1-mediated p38 alternative activation negatively regulates the activities of caspases and Gadd45α in Con A-induced acute liver injury (Fig. 7E). Taken together with our previous study about the role of classical p38 activation in liver, our results indicate a protective role for p38 in liver during inflammatory diseases and development of strategies that deliver inhibitors specifically to inflammatory cells but not to hepatocytes may provide benefit for the treatment of inflammatory diseases without the liability of severe liver inflammation.

## Methods

### Mice and acute liver injury model

C57BL/6J background wild–type and p38αβ^Y323F^ (Stock No: 012566) mice (males and females, 6–8 weeks-old) were obtained from the Jackson Laboratory. Protocols for the use of animals were approved by the Institutional Animal Care and Use Committee (IACUC) and animal experiments were conducted in accordance with the humane care guidelines at The Scripps Research Institute. To induce acute liver injury in mice, Concanavalin A (20 mg/kg body weight in PBS) was administered intravenously via the lateral tail vein^9^. Mice were euthanized, blood was collected, and livers were surgically removed.

### Generation of bone marrow chimeras

Chimeric mice were generated by total-body gamma irradiation followed by transfer of bone marrow cells^35^. Briefly, WT and p38YF mice were irradiated with a dose of 10 Gy and then BM cells from WT and p38YF mice were intravenously injected (1 × 10^6^ cells per mouse). After 5–6 weeks, liver injury was induced by an intravenous injection of Con A.

### Cell culture

Mouse hepatocyte Hepa 1–6 cells were cultured in DMEM supplemented with 10% FBS, and human hepatocellular carcinoma HepG2 cells were cultured in EMEM supplemented with 10% FBS. Cell viability was determined by XTT assay (Roche).

### Reagents

Concanavalin A, actinomycin D and XTT (2,3-Bis(2-methoxy-4-nitro-5-sulfophenyl)-2*H*-tetrazolium-5-carboxanilide inner salt) were obtained from Sigma-Aldrich; mouse or human TNF-α was from PeproTech; α-galactosyl ceramide (α-GalCer; KRN7000) was from Enzo Life Science; R788 was from Cayman Chemical.

Antibodies to IκBα, p-ERK, p-JNK, p-p38 (T180Y182), p-MKK3/6, p-MKK4, SYK, PLCγ1, Vav-1, SLP-76, PKCθ, cleaved caspase 3, caspase 9, and cleaved caspase 9 were purchased from Cell Signaling Technology; anti-p-p38 (Y323) was from ThermoFisher Scientific; anti-GAPDH Ab was from Chemicon; Abs to mouse CD3, CD28, and Zap70 were from Biolegend; anti-CD3-FITC, anti-CD4-PE, anti-NK 1.1-PE, and anti-CD69-PerCP Abs, and Annexin V-FITC apoptosis detection kit were from eBioscience; anti-Gadd45α Ab was from Santa Cruz Biotechnology.

### ALT and AST measurement

Serum ALT and AST levels were measured using assay kits from Pointe Scientific.

### Histology

Liver tissues were fixed in 10% neutral buffered formalin and processed for H&E staining. TUNEL staining was performed using *In Situ* Cell Death Detection Kit (Roche).

### Quantitative PCR

Total RNA from liver or cells was prepared with TRIzol reagent (Invitrogen). cDNA templates were prepared by using Superscript IV reverse transcriptase (Invitrogen). Quantitative PCR analysis was performed in a BioRad CFX cycler using Power SYBR Green Master Mix (Thermo Fisher). Following primers were used: 5′-AAGGTCATCCCAGAGCTGAA-3′ and 5′-CTGCTTCACCACCTTCTTGA-3′ for *gapdh*; 5′- GGCCATCAGCAACAACATAAGCGT-3′ and 5′-TGGGTTGTTGACCTCAAACTTGGC-3′ for *ifng*; 5′-ATGAGAAGTTCCCAAATGGC-3′ and 5′-CTCCACTTGGTGGTTTGCTA-3′ for *tnfa*. Expression level of *gapdh* mRNA was measured to normalize the mRNA levels of genes.

### Hepatocyte preparation

Livers were perfused and digested by passing warm perfusion buffer (HBSS supplemented with 10 mM HEPES and 0.5 mM EGTA) and digestion buffer (HBSS supplemented with 10 mM HEPES and 4.12 μg/ml liberase) sequentially via the portal vein. Liver tissue was further gently digested, and cells were passed through cell strainers. Hepatocytes were isolated by centrifugation at 40 × g for 1 min at RT, and the cell pellet was washed with PBS three times. Purified hepatocytes were resuspended in William E medium (Quality Biological, Inc.) supplemented with 10% FBS and antibiotics, seeded on collagen-coated culture plates (50 μg/ml, Stem Cell Technologies) for 1 h at RT, and then washed with PBS twice before use in experiments^9,35^.

### Preparation of liver leukocytes

HBSS was injected through the portal vein to remove blood from the liver. Liver homogenates were incubated with 100 U/ml collagenase (Sigma) for 40 min at 37 °C, and the debris was removed by passing the digest through a 100-μm cell strainer. Cells were centrifuged at 700 × g for 10 min at RT, and the pellets were resuspended in 40% isotonic Percoll containing 100 U/ml of heparin. The suspension was centrifuged at 700 × g for 20 min at RT. Liver leukocytes were stimulated with plate-bound anti-CD3 (10 μg/ml) and anti-CD28 (2 μg/ml), or α-GalCer (100 ng/ml)^9,35^.

### Plasmids and short hairpin RNAs

Flag-tagged WT p38α-expressing mammalian expression vector was obtained from Jiahuai Han, and substitution of Tyr at 323 to Phe (Y323F) was generated by site-directed mutagenesis using the QuikChange kit (Stratagene). Hepa 1-6 cells expressing WT p38 or mutant p38YF were generated by transfecting the plasmids using Lipofectamine 2000 (Invitrogen).

Lentiviral vectors expressing the short hairpin RNAs (shRNAs) that target the genes were obtained from Sigma (MISSION^®^ TRC shRNAs). Lentivirus packaging and transduction was according to the manufacturer’s instructions. Lentivirus titer was determined with a Lenti-X p24 ELISA (Clontech, Mountain View, CA, USA).

### Immunoblotting

Liver tissues or hepatocytes were incubated in lysis buffer supplemented with protease inhibitors to prepare the lysates. Tissue or cell lysates were resolved by SDS-PAGE and analyzed by immunoblotting using chemiluminescence substrate.

### Measurement of cytokines

TNF-α or IFN-γ concentrations in sera or culture supernatants were measured by ELISA (eBioscience).

### Cell death analysis by flow cytometry

WT or YF Hepa 1–6 cells (2.5 × 10^5^ cells/ml) were incubated with actinomycin D (1 μg/ml) and TNF-α. Cells were harvested and stained with Annexin V-FITC and propidium iodide (PI) (Invitrogen), and cell death was analyzed using LSR-II flow cytometer (Becton Dickinson).

### Statistical analysis

Statistical significance was determined by Student’s t test for comparing two groups or one-way ANOVA followed by Dunnet post hoc test for comparing multiple groups using Prism software (GraphPad). *P* < 0.05 was considered to be statistically significant.

## Supplementary information

Supplementary Information

## Data Availability

The datasets generated during and/or analyzed during the current study are available from the corresponding author on reasonable request.
