# Supplementary material for: The protein tyrosine kinase SYK regulates the alternative p38 activation in liver during acute liver inflammation
Source: Sci Rep. 2019 Nov 28;9:17838. doi: 10.1038/s41598-019-54335-3 (PMC6882802; doi:10.1038/s41598-019-54335-3)
Supplement: Supplementary file 1 — Supplementary Information [file 41598_2019_54335_MOESM1_ESM.pdf]

The protein tyrosine kinase SYK regulates the alternative p38 activation in liver during acute liver inflammation.

Bo-Ram Bang[a,c], Kyung Ho Han[b], Goo-Young Seo[d], Michael Croft[e], and Young Jun Kang[a,f, *]

a. Department of Immunology and Microbial Science, The Scripps Research Institute, La Jolla, CA 92037, USA,
b. Department of Chemistry, The Scripps Research Institute, La Jolla, CA 92037, USA,
c. Department of Medicine, Division of Gastrointestinal and Liver Diseases, Keck School of Medicine, University of Southern California, Los Angeles, CA 90033, USA,
d. Division of Developmental Immunology, La Jolla Institute for Immunology, La Jolla, CA 92037, USA
e. Division of Immune Regulation, La Jolla Institute for Immunology, La Jolla, CA 92037, USA,
f. Molecular Medicine Research Institute, Sunnyvale, CA 94085, USA

***Correspondence**
Young Jun Kang,
Molecular Medicine Research Institute,
428 Oakmead Parkway, Sunnyvale, CA 94085, USA
Tel; (408) 523-6260
E-mail; ykang@mmrx.org

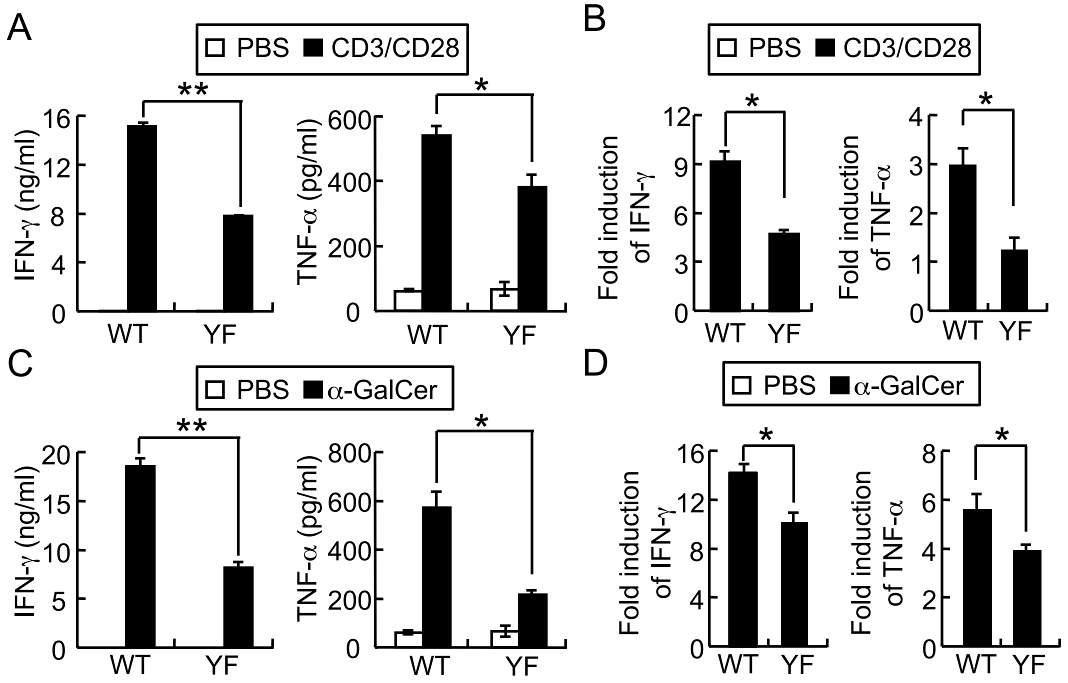

Figure S1. Expression of inflammatory cytokines is regulated by alternative p38 signaling in T and NKT cells. Liver leukocytes of WT or p38YF mice were treated with PBS or anti-CD3/CD28 Abs (A & B), or PBS or α-GalCer (C & D). (A & C) Culture supernatants were obtained after 24 h to determine IFN-γ and TNF-α concentrations by ELISA. (B & D) Total RNAs were prepared after 4 h of stimulation and the expression of cytokines was measured by qPCR. Fold induction of genes were normalized the levels of the unstimulated cells. Data shown are means ± s.d.; *$p < 0.05$ and **$p < 0.01$. Results shown are representative of 2-3 independent experiments.

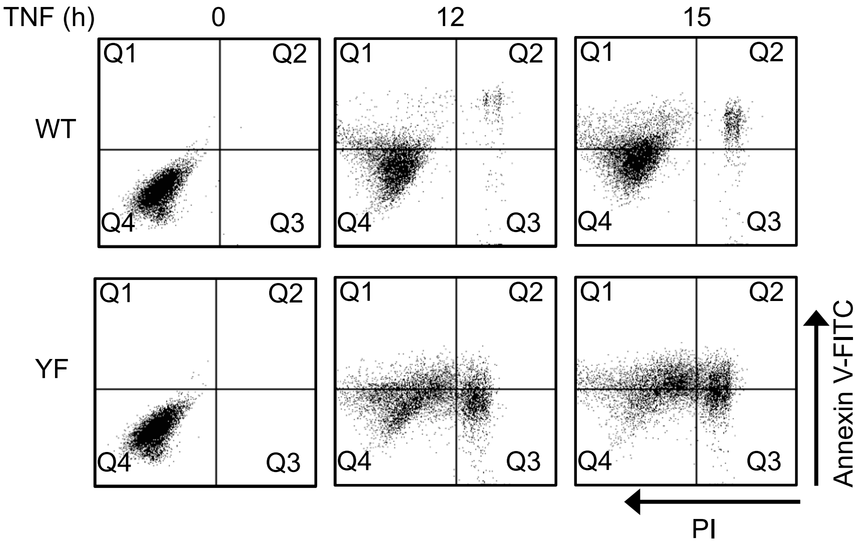

Cell types	TNF (h)	Q1	Q2	Q3	Q4
WT	0	0.24 ± 0.06	0.02 ± 0.00	0.02 ± 0.01	99.70 ± 0.10
YF		0.04 ± 0.05	0.03 ± 0.03	0.03 ± 0.04	99.80 ± 0.10
WT	12	27.23 ± 3.50	3.92 ± 0.59	1.46 ± 0.91	67.39 ± 3.36
YF		32.00 ± 2.00	9.01 ± 1.48	19.33 ± 1.66	39.65 ± 0.31
WT	24	29.80 ± 1.70	6.37 ± 1.05	1.91 ± 0.54	62.40 ± 2.10
YF		31.40 ± 6.80	13.88 ± 4.02	22.65 ± 4.35	32.08 ± 6.47

Figure S2. Flow cytometry analysis of cell death. WT or p38YF hepatocytes were incubated with ActD (1 μg/ml) and TNF (0 or 30 ng/ml) for 0, 12 or 15 h. Cells were incubated with Annexin V-FITC and PI, and cell death was analyzed by flow cytometry (n = 3). Percentage of each quadrant is shown in the table. Data represent means ± s.d. Results shown are the representative of 2 independent experiments.

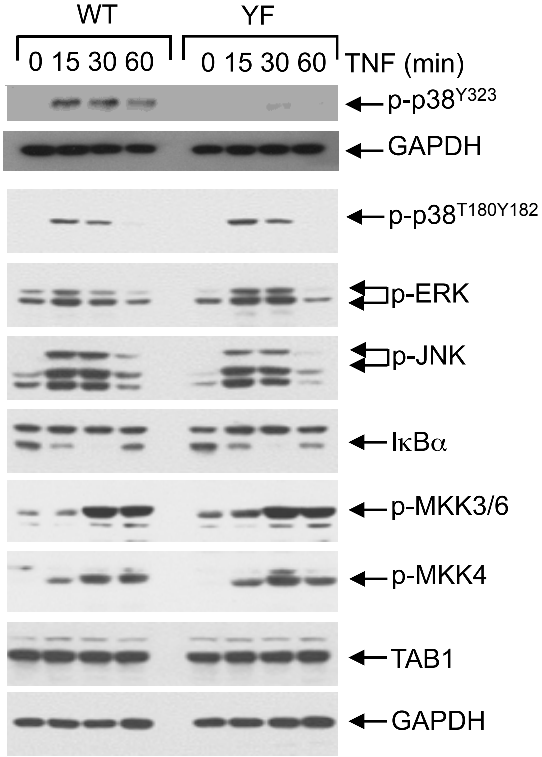

Figure S3. Alternative p38 activation is not regulated by the MKK-mediated signaling pathway. WT or p38YF Hepa 1-6 cells were treated with TNF and activation of signaling molecules were determined by immunoblotting using the indicated Abs. GAPDH levels were examined as an internal loading control. Data represent means ± s.d.; $*p < 0.05$, and N.S., not significant. Results shown are the representative of 3 independent experiments. Full-length blot images are shown in the supplementary information (Fig. S10).

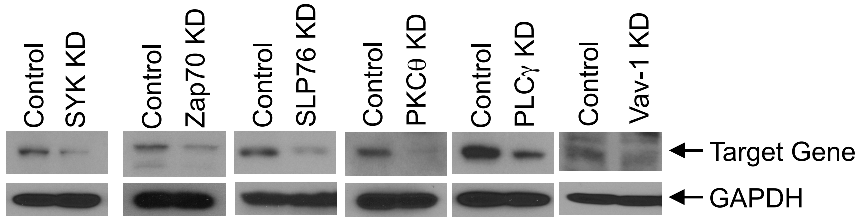

Figure S4. Knockdown of SYK, Zap70, SLP76, PKCθ, PLCγ or Vav-1. Hepa 1-6 cells were infected with lentiviruses encoding control (scramble), SYK, Zap70, SLP76, PKCθ, PLCγ or Vav-1 shRNAs. KD of target genes was analyzed by immunoblotting using antibodies to each protein. GAPDH levels were determined as a loading control. Full-length blot images are shown in the supplementary information (Fig. S11&12).

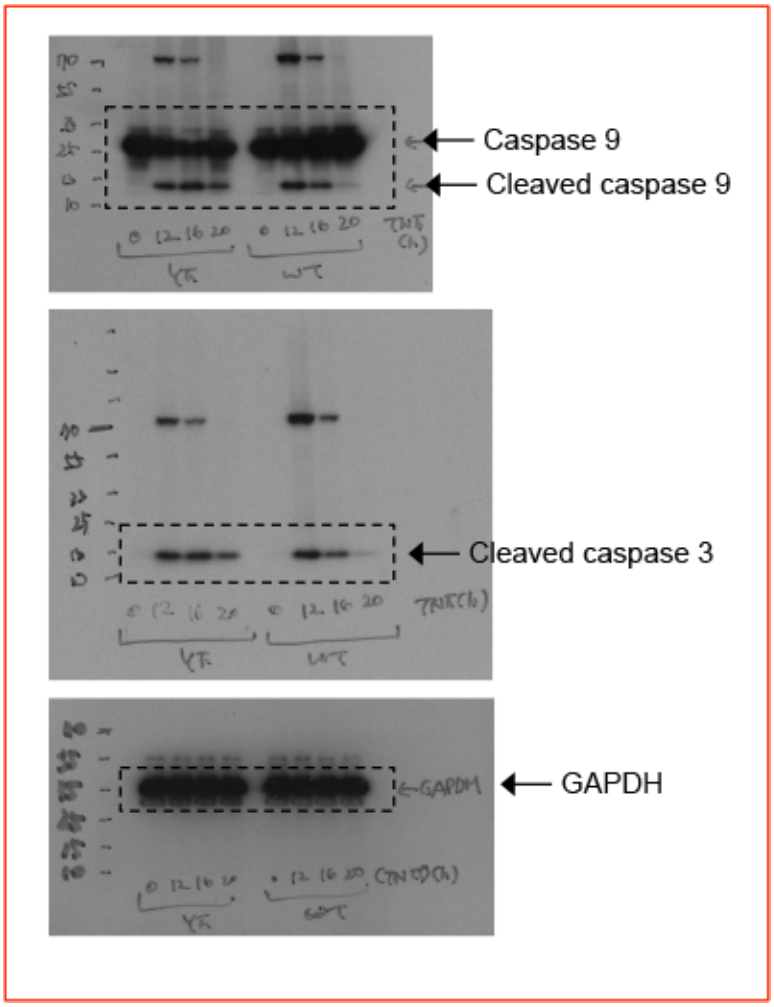

Fig. S5. Uncropped images of Figure 4C

Uncropped images of Figure 5B

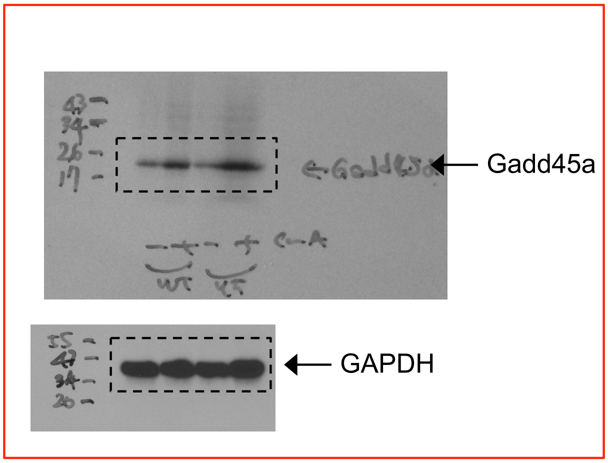

Uncropped images of Figure 5C

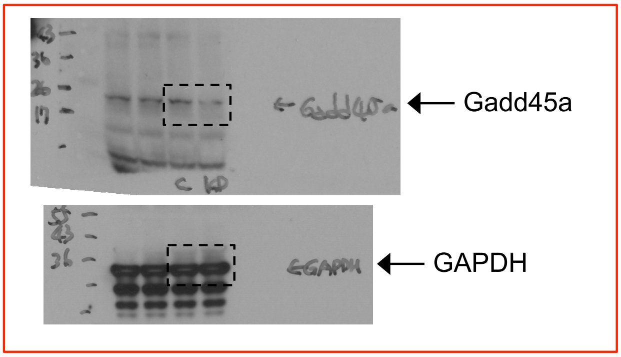

Fig. S6. Uncropped images of Figure 5 B & C

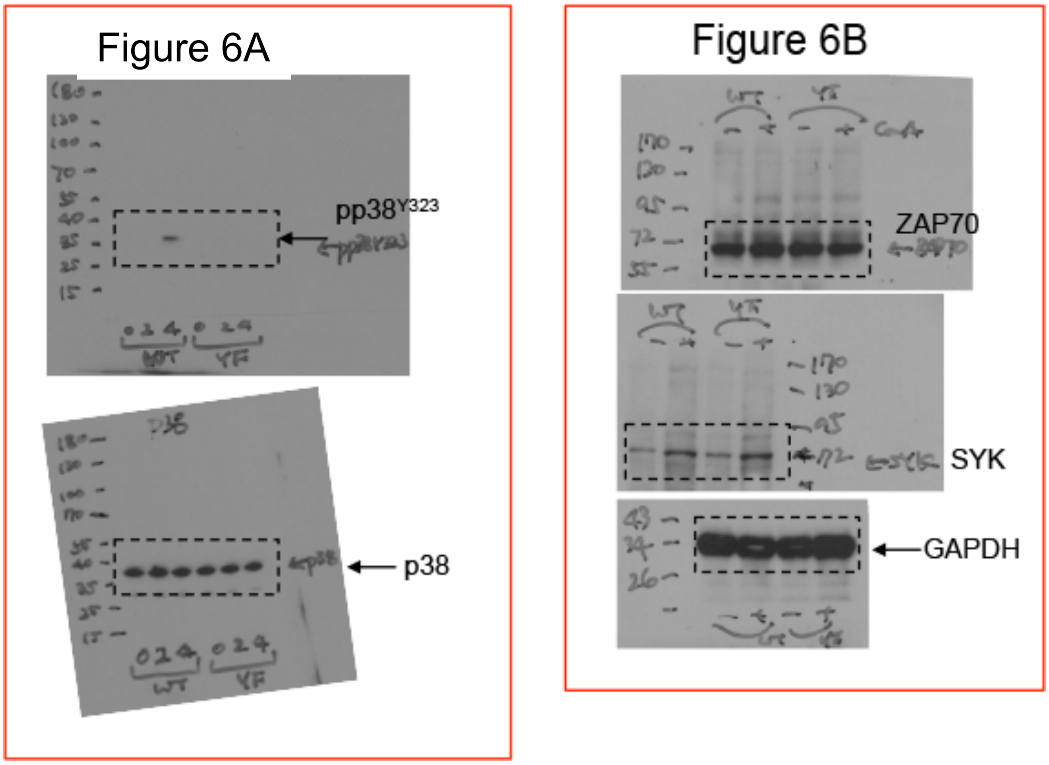

Fig. S7. Uncropped images of Figure 6 A&B

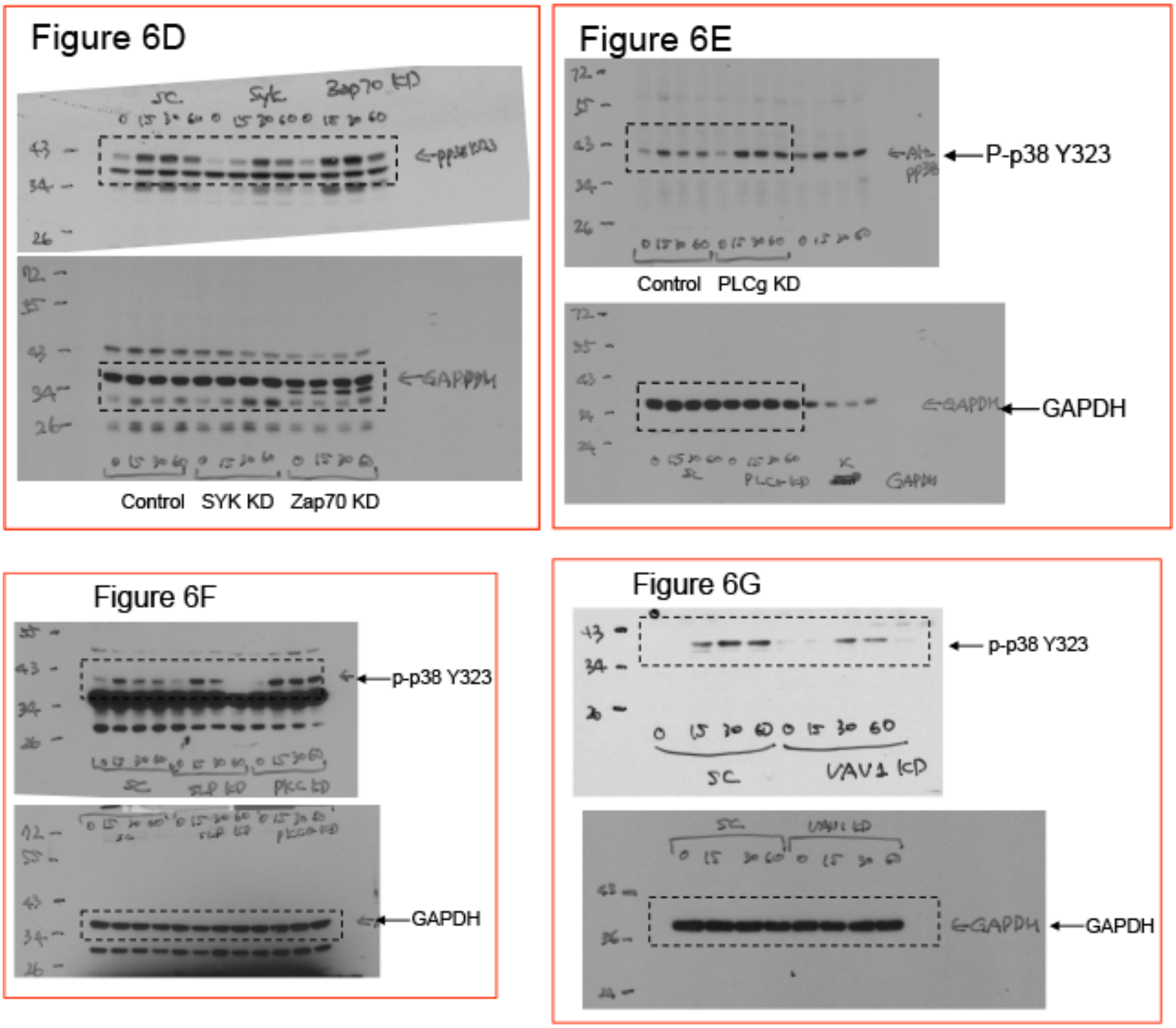

Fig. S8. Uncropped images of Figure 6 D-G

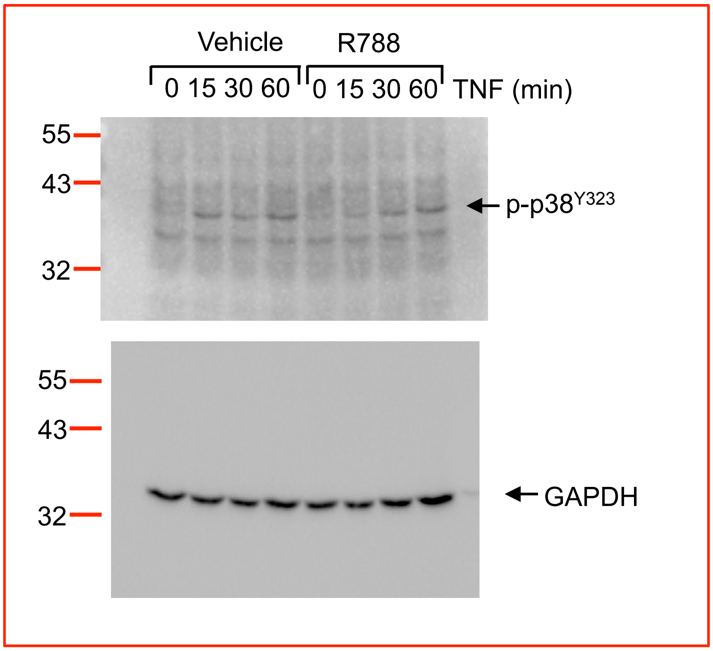

Fig. S9. Uncropped images of Figure 7B

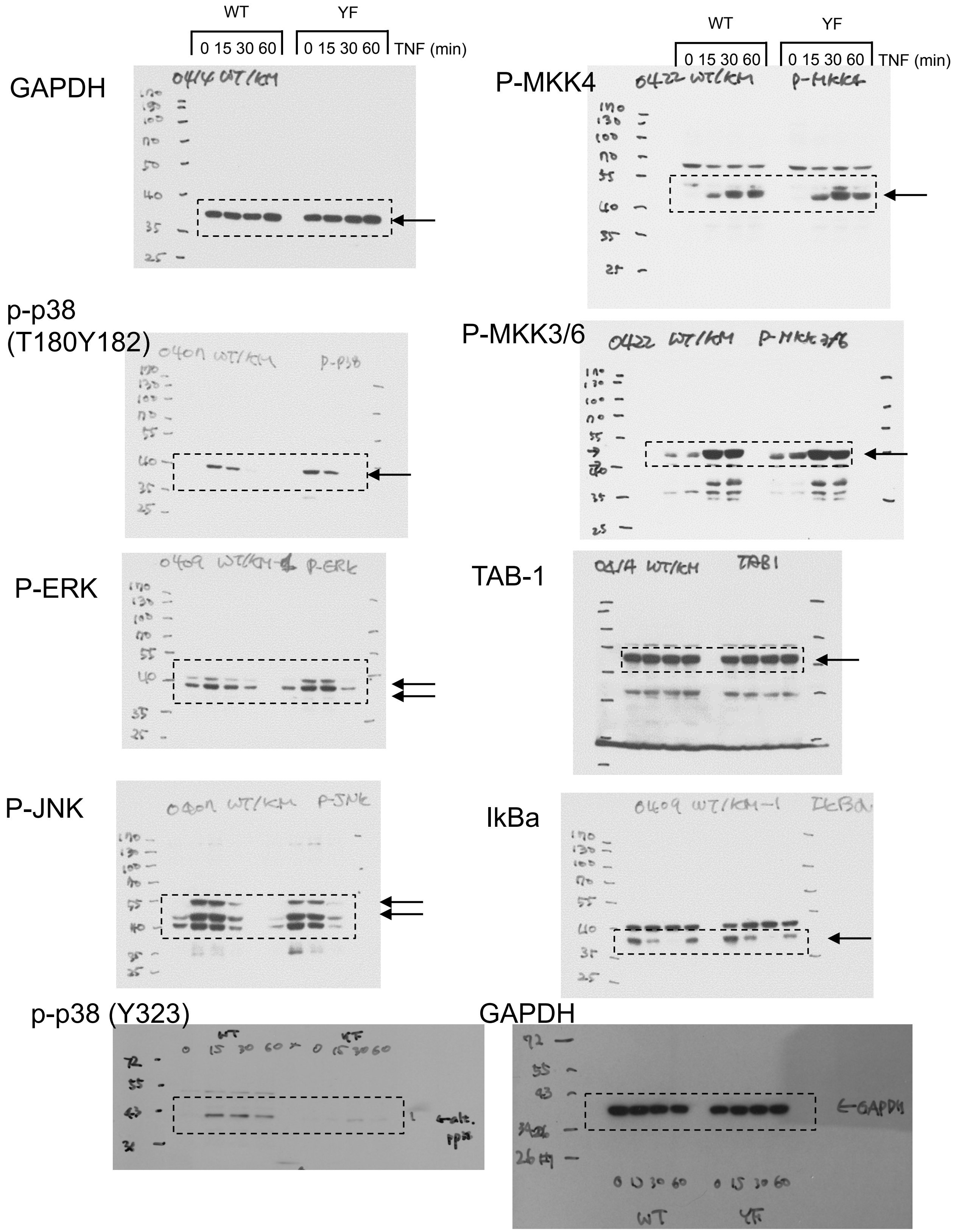

Fig. S10. Uncropped images of Figure S3

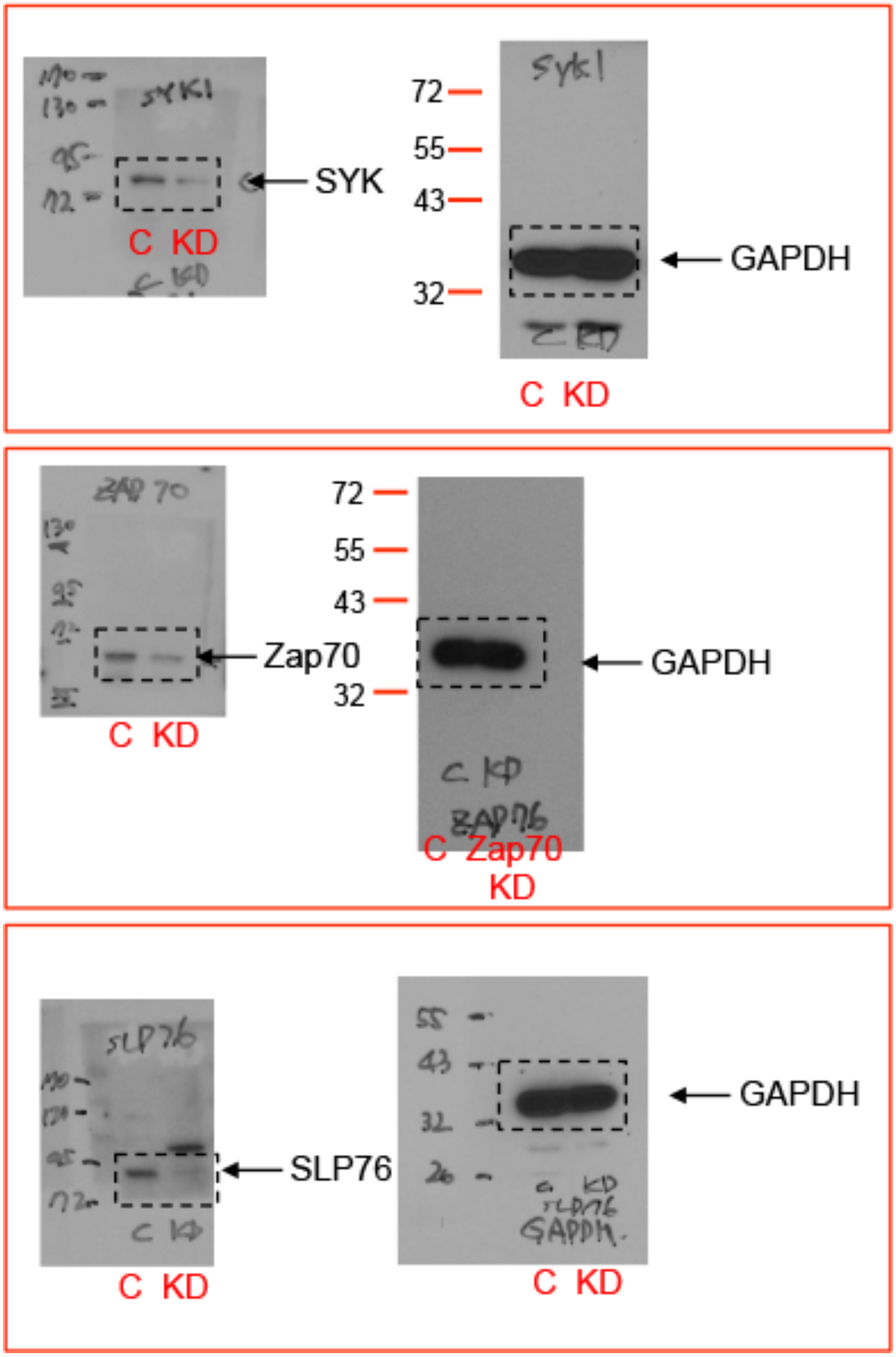

Fig. S11. Uncropped images of Figure S4

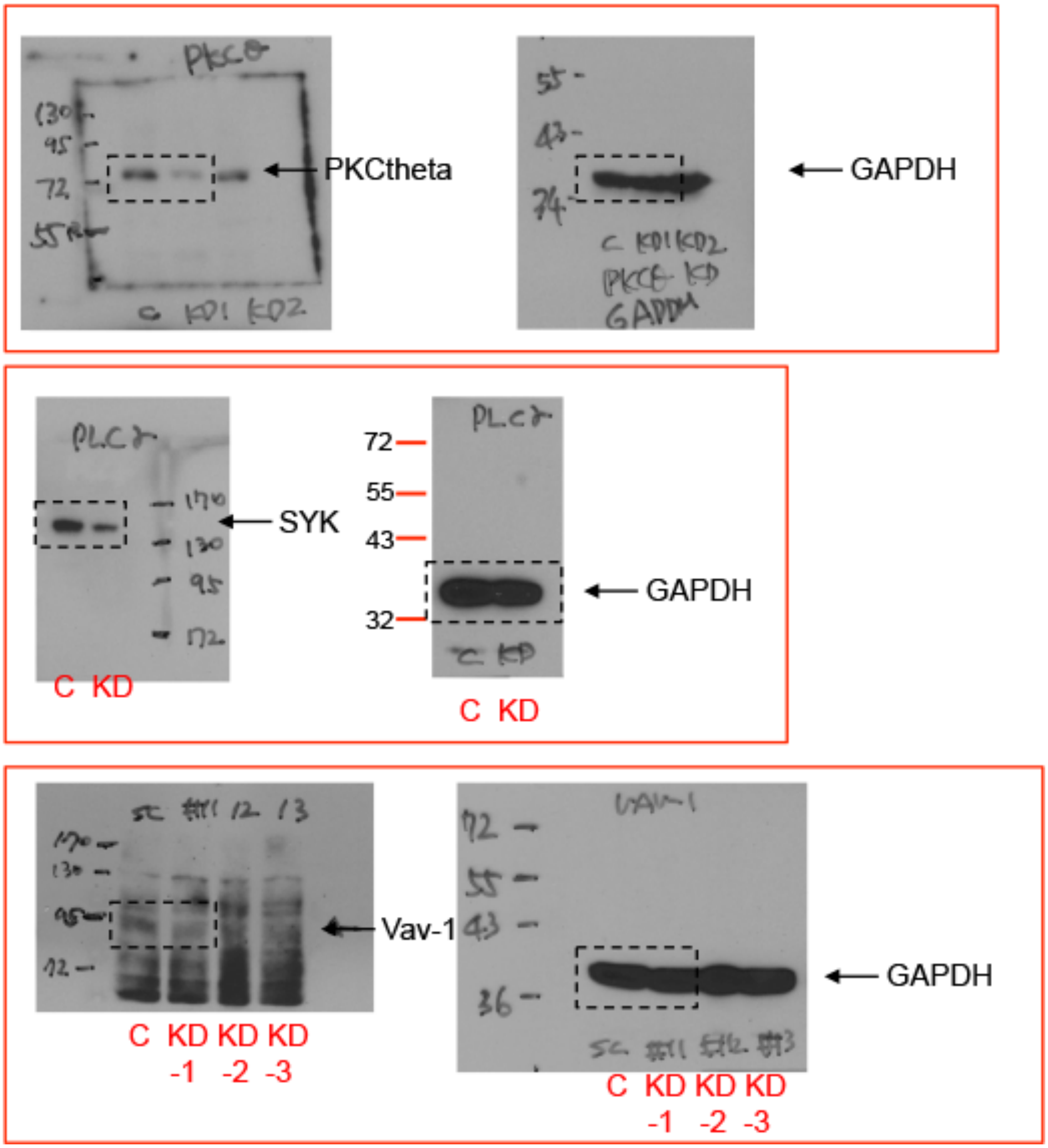

Fig. S12. Uncropped images of Figure S4
